# Skeletal standardized uptake values obtained using quantitative SPECT/CT for the detection of bone metastases in patients with lung adenocarcinoma

**DOI:** 10.3389/fmed.2023.1119214

**Published:** 2023-02-02

**Authors:** Lin Lin, Rong Zheng, Jianhua Geng, Xuejuan Wang, Meng Li, Rong Fan, Yiqing Zheng, Ke Yang

**Affiliations:** ^1^Department of Nuclear Medicine, National Cancer Center/National Clinical Research Center for Cancer/Cancer Hospital, Chinese Academy of Medical Sciences and Peking Union Medical College, Beijing, China; ^2^Department of Radiology, National Cancer Center/National Clinical Research Center for Cancer/Cancer Hospital, Chinese Academy of Medical Sciences and Peking Union Medical College, Beijing, China

**Keywords:** Tc-99m methylene-diphosphonate, quantitative single-photon emission computed tomography/computed tomography, standardized uptake value, lung adenocarcinoma, bone metastases

## Abstract

**Purpose:**

To assess the utility of skeletal standardized uptake values (SUVs) obtained using quantitative single-photon emission computed tomography/computed tomography (SPECT/CT) in differentiating bone metastases from benign lesions, particularly in patients with lung adenocarcinoma.

**Methods:**

Patients with lung adenocarcinoma who had undergone whole-body Tc-99m methyl-diphosphonate (^99m^Tc-MDP) bone scans and received late phase SPECT/CT were retrospectively analyzed in this study. The maximum SUV (SUVmax); Hounsfield units (HUs); and volumes of osteoblastic, osteolytic, mixed, CT-negative metastatic and benign bone lesions, and normal vertebrae were compared. Receiver operating characteristic curves were used to determine the optimal cutoff SUVmax between metastatic and benign lesions as well as the cutoff SUVmax between CT-negative metastatic lesions and normal vertebrae. The linear correlation between SUVmax and HUs of metastatic lesions as well as that between SUVmax and the volume of all bone lesions were investigated.

**Results:**

A total of 252 bone metastatic lesions, 140 benign bone lesions, and 199 normal vertebrae from 115 patients with lung adenocarcinoma were studied (48 males, 67 females, median age: 59 years). Metastatic lesions had a significantly higher SUVmax (23.85 ± 14.34) than benign lesions (9.67 ± 7.47) and normal vertebrae (6.19 ± 1.46; *P* < 0.0001). The SPECT/CT hotspot of patients with bone metastases could be distinguished from benign lesions using a cutoff SUVmax of 11.10, with a sensitivity of 87.70% and a specificity of 80.71%. The SUVmax of osteoblastic (29.16 ± 16.63) and mixed (26.62 ± 14.97) lesions was significantly greater than that of osteolytic (15.79 ± 5.57) and CT-negative (16.51 ± 6.93) lesions (*P* < 0.0001, *P* = 0.0003, and 0.002). SUVmax at the cutoff value of 8.135 could distinguish CT-negative bone metastases from normal vertebrae, with a sensitivity of 100.00% and a specificity of 91.96%. SUVmax showed a weak positive linear correlation with HUs in all bone metastases and the volume of all bone lesions.

**Conclusion:**

SUVmax of quantitative SPECT/CT is a useful index for distinguishing benign bone lesions from bone metastases in patients with lung adenocarcinoma, particularly in the diagnosis of CT-negative bone metastases, but other factors that may affect SUVmax should be considered.

## 1. Introduction

According to the International Agency for Research on Cancer (IARC) 2020 global burden of cancer statistics, lung cancer ranks second in global incidence and first in mortality (1). Lung cancer remains the most common cancer type in China and the leading cause of cancer-related deaths (2). Adenocarcinoma has become the most common subtype of lung cancer, with increasing prevalence (3). The skeleton is one of the most common metastatic sites in patients with advanced lung cancer, with an incidence of bone metastasis of 30%−40% (4). Unlike the mostly osteoblastic bone metastases of prostate cancer, the bone metastases of lung cancer may include osteolytic, osteoblastic, mixed, and CT-negative metastases and may exhibit complex CT features. Therefore, it is more challenging to diagnose bone metastases in patients with lung cancer. We here in focused on patients with lung adenocarcinoma, who account for the vast majority of lung cancer cases in China (2, 3).

Bone scintigraphy (BS) is one of the most commonly used methods for early screening and detection of bone metastases in the whole skeleton (5, 6). However, with low regional blood flow and osteogenic activity as well as low spatial resolution, it is relatively insensitive for detecting changes in bone metastatic tumors. Furthermore, some benign lesions can produce a false positive signal during BS evaluation, thereby limiting the specificity of this imaging technique (7). Single-photon emission computed tomography/computed tomography (SPECT/CT) enables characterization of morphological changes and determination of anatomical correlations and attenuation corrections of radiotracer uptake on CT, resulting in a significant improvement in diagnostic accuracy, particularly when assessing indeterminate lesions on planar BS (8). Studies have shown that adding SPECT/CT to BS improves the specificity, positive predictive value, and diagnostic confidence of the reader, thereby reducing the number of equivocal study reports (9, 10). SPECT has conventionally been used as a nonquantitative method; however, wide acceptance of integrated SPECT/CT scanners and development of iterative reconstruction algorithms have made it possible to use quantitative SPECT in clinical settings (11–13).

Quantitative SPECT and positron emission tomography (PET) enable the calculation of standardized uptake values (SUVs), which can be used for disease assessment and interpatient comparisons (14–17). In a previous study, a strong correlation between the SUVs of ^99m^Tc-hydroxyethylene diphosphonate (HDP) SPECT/CT and those of ^18^F-NaF PET/CT demonstrated that SPECT is an applicable tool for clinical quantification of bone metabolism in osseous metastases in patients with breast and prostate cancer (18). Furthermore, SUVs can be used to broaden the visual analysis of skeletal structures (18). Previous studies on patients with prostate cancer showed that the SPECT SUVmax of bone metastases was significantly higher than that of benign bone lesions, degenerative joint disease of bone, and benign spinal and pelvic osteoarthritic changes (14, 16, 17, 19). Because the CT manifestations of bone metastases in patients with lung cancer are more complex, the SUVmax results of quantitative SPECT/CT are highly variable among different types of bone lesions in these patients (15, 20). As the feature of SUVmax in patients with lung cancer is quite different from that in patients with prostate cancer, a thorough investigation is necessary. However, few studies have performed quantitative SPECT/CT bone imaging analysis of the SUVmax of bone metastases in patients with lung adenocarcinoma.

This study aimed to analyze the SUVmax levels of bone metastases of four different CT features obtained using quantitative SPECT/CT in patients with lung adenocarcinoma as well as the SUVmax cutoff values to distinguish bone metastases from benign lesions and the SUVmax cutoff values for distinguishing CT-negative bone metastases from normal vertebrae. We also investigated the effect of the volume of lesions and Hounsfield units (HUs) on SUVmax.

## 2. Materials and methods

### 2.1. Patients

A total of 115 patients with lung adenocarcinoma who had undergone bone scans and SPECT/CT at the Cancer Hospital, Chinese Academy of Medical Sciences, and Peking Union Medical College from September 2021 to May 2022 were analyzed. The following criteria were used to determine inclusion: (i) bone scans and SPECT/CT performed on the same day; (ii) no receipt of treatment for skeletal metastatic lesions prior to imaging analyses; and (iii) no history of other primary malignancies. The exclusion criteria were as follows: (i) no available bone scan or SPECT/CT results; (ii) patients who were lost to follow-up; and (iii) no definite histopathological diagnosis of the primary lesion.

All patients were followed up for at least 6 months (10.7 ± 2.3, 6.0–14.7 months). For ethical and practical reasons, biopsy-based confirmation of patient bone metastases was not performed; instead, the final diagnosis of these metastases was based on a combination of imaging examination results (BS, CT, MRI, or PET/CT) and clinical follow-up (physical signs and follow-up imaging examinations).

The Ethics Committee of the Cancer Hospital, Chinese Academy of Medical Sciences, and Peking Union Medical College approved this study, which followed the 1964 Helsinki Declaration ethical standards and its subsequent amendments. All patients provided written informed consent.

### 2.2. Image acquisition

All patients underwent whole-body planar imaging (scanned 2.5–4.5 h after injection)with a low-energy high-resolution collimator and quantitative SPECT/CT (Siemens Symbia Intevo 6, USA) on planar scintigraphy high-uptake regions. SPECT was acquired at a mean patient dose of 831 ± 44 MBq (22.45 ± 1.19 mCi, range: 20.00–25.20 mCi) ^99m^Tc-MDP intravenous injection (from HTA Co., Ltd., and Beijing Senke Pharmaceutical Co., Ltd) and 0–3.5 h after the whole-body planar bone scan. Images were captured using a 256 × 256 matrix size and 6 degrees rotation/step, 15 seconds/projection. CT scans were performed using adaptive dose modulation at 130 kV and 60 mAs (Siemens Care Dose). The CT data were reconstructed using B60s medium sharp with a slice thickness of 2.5 mm. SPECT images were reconstructed using the Flash 3D algorithm (xSPECT Skeletal mode) with eight iterations, four subsets, and a Gaussian filter. The SPECT reconstructed values were decay-corrected to the time of injection and final values of quantitative radioactivity concentrations were obtained to allow SUV body weight quantification (SUVbw) on post-processed images and measurement of SUVmax (g/ml) using the xSPECT reconstruction algorithm. SUVmax was defined as the pixel value with the highest activity concentration within a volume of interest (VOI).

### 2.3. Image interpretation

The ^99m^Tc-MDP planar and SPECT/CT bone scans were interpreted independently by two nuclear medicine physicians who were blinded to the clinical history and findings of each other. The reviewers had 10 and 8 years of experience in nuclear medicine, respectively. The combination of SPECT and CT images was analyzed first, followed by a planar whole-body bone scan. Disagreements in lesion interpretation were resolved through consensus and joint reading.

For SPECT/CT fusion imaging analysis, bone metastases were diagnosed if the CT revealed osteolytic changes (bone erosion, edge irregularity, no osteosclerosis, or a soft-tissue mass), osteoblastic changes (high bone density without a soft-tissue mass) in areas of abnormal radioactivity concentrations. If an abnormal radioactivity concentration was observed involving the centrum or pedicle of the vertebral arch or another part of the skeleton but the CT did not show eroded bone damage or a soft-tissue mass, an early-stage CT-negative metastatic bone diagnosis was made. A benign lesion was diagnosed if the CT revealed degenerative changes, such as hyperosteogeny, osteosclerosis, osteophytes, Schmorl's nodes, a bone island, or a fracture in the lesion area with an abnormal radioactivity concentration (14).

**Figure 1:**
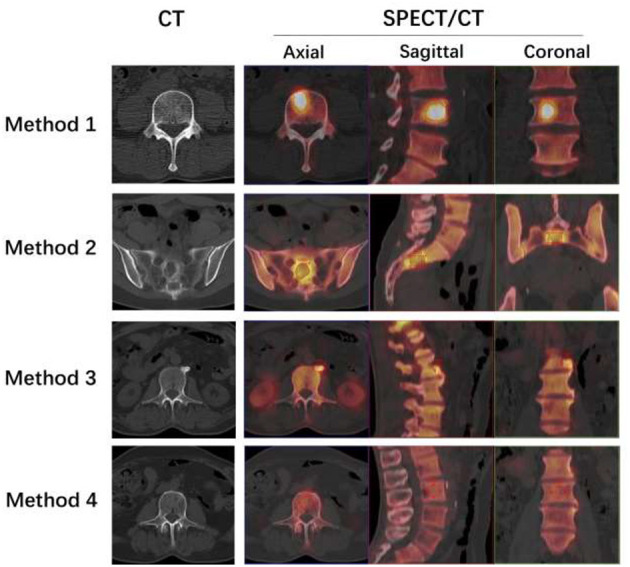
Four methods to delineate different VOIs. **Method 1**: The L4 bone metastatic lesion was delineated by putting the tumor VOI at 40% of the SUVmax on the SPECT/CT fusion images of axial, sagittal, and coronal sections. The axial section of CT showed no abnormal density changes. **Method 2**: The Siemens “Multi-frame Polygon” tool was used to manually draw an osteolytic bone metastatic lesion of the sacrum on axial SPECT/CT fusion sections from the top to the bottom of the lesion. Then, the sagittal and coronal images were automatically delineated. The axial section of CT showed osteolytic bone destruction of sacrum. **Method 3**: On axial, sagittal, and coronal SPECT/CT fusion sections, the hyperosteogeny lesion of L2 was delineated using the “Ellipsoid” tool. The axial section of CT showed osteophytes of L2. **Method 4**: The visually normal L3 vertebral body was delineated on axial, sagittal, and coronal SPECT/CT fusion sections with volumes not more than 5 cm^3^ using the “Ellipsoid” tool, excluding the bone cortex. The axial section of CT showed normal vertebrae of L3.

Using “Siemens 3D Isocontour,” hotspot lesions were drawn on transversal, sagittal, and coronal SPECT/CT fusion sections by placing the VOI with the margin threshold set at 40% of the SUVmax (Figure 1: Method 1). The Siemens “Multi-frame Polygon” tool was used to manually draw osteolytic bone lesions on transversal SPECT/CT fusion sections (Figure 1: Method 2). The VOIs of no more than three of the largest bone metastatic lesions visible on SPECT were drawn for patients with multiple metastases. On transversal, sagittal, and coronal SPECT/CT fusion sections, hyperosteopathic lesions or other benign lesions with relatively low uptake were delineated using the Siemens “Ellipsoid” tool (Figure 1: Method 3). On transversal, sagittal, and coronal SPECT/CT fusion sections with volumes ranging from 1 to 5 cm^3^, visually normal spinal vertebral body was delineated using the “Ellipsoid” tool, excluding the bone cortex (Figure 1: Method 4). Normal vertebrae were chosen from cervical, thoracic, and lumbar vertebrae, with one for each in the scan field. Bone density values were measured in HUs on the CT images of SPECT/CT.

### 2.4. Statistical analysis

SPSS software V.22.0 (IBM SPSS) and GraphPad Prism 8.0 were used for statistical analyses. All statistical data are presented as mean ± standard deviation (SD). Mann–Whitney nonparametric test was used to compare median values between two unpaired groups. Kruskal–Wallis test for independent samples was used to compare median values among three or more unpaired groups. Receiver operating characteristic (ROC) curve analysis was used to determine the best SUVmax cutoff value. A *P*-value of < 0.05 was considered statistically significant for all tests. Linear correlations between SUVmax and HUs and between SUVmax and volume were analyzed.

## 3. Results

### 3.1. Distribution of all lesions and types of benign bone lesions

A total of 252 bone metastatic lesions were analyzed from 115 patients with lung adenocarcinoma (Table 1 shows the characteristics of the patients). Of the 252 metastatic lesions, 123 were located in the spine (cervical, thoracic, and lumbar), 38 in the thorax (including the ribs, clavicle, sternum, and scapula), 75 in the pelvis (including the hip, sacrum, and sacroiliac region), 15 at the limbs, and one in the skull. Among the 140 benign bone lesions, 89 were present in the spine, 12 in the thorax, 25 in the pelvis, and 14 in the limbs. Among the 199 normal vertebrae, 20 were cervical vertebrae, 90 thoracic vertebrae, and 89 lumbar vertebrae.

**Table 1:** Participant characteristics.

**Characteristic**	**Value**
Mean age (years)^*^	58.38 ± 9.92 (34–78)
**Gender**	
Men (*n*, %)	48 (41.7%)
Women (*n*, %)	67 (58.3%)
**Metastatic lesions (** ***n*** **)** ^†^	
0	10
1–5	58
6–10	19
11–20	18
>20	10

* Data are the mean ± standard deviation; data in parentheses are the range.

† The data are patient numbers in the various metastatic number ranges listed below.

Among the 140 benign bone lesions, 107 lesions (76.4%) were hyperosteogeny (including 82 osteophytes, 13 hyperplastic sclerosis of the sacroiliac joint, and 12 hyperplasia of the sternoclavicular or costal vertebra joint); 12 lesions (8.6%) were single focal lesions that occurred in the iliac bone or in the proximal femur or humerus, showing a clear boundary of sclerosis (may be bone infarct, bone cyst, fibrous dysplasia of bone, or other benign bone lesions); four lesions (2.9%) were osteitis; four lesions (2.9%) were vertebral hemangiomas; three lesions (2.1%) were compacta bone islands; three lesions (2.1%) were bone fractures; three lesions (2.1%) were the Schmorl's nodes; two lesions (1.4%) may have been enchondromas; one lesion (0.7%) may have been fibrous dysplasia of bone; and one lesion (0.7%) may have been a vasculogenic lesion of fibula.

### 3.2. SUVmax, HUs, and volume differences between metastatic and benign bone lesions and normal vertebrae

The SUVmax of metastatic lesions (23.85 ± 14.34) was higher than that of benign lesions (9.67 ± 7.47) and normal vertebrae (6.19 ± 1.46). The difference among the SUVmax of the three groups was statistically significant (*P* < 0.0001).

**Figure 2:**
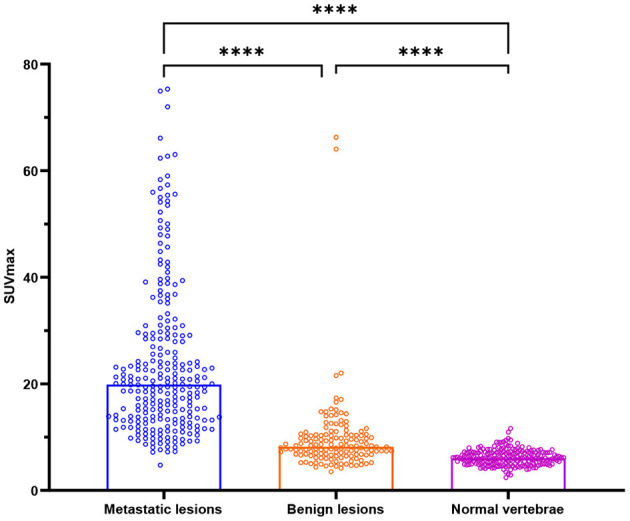
Box plots of all lesions in relation to the SUVmax (g/ml) for bone metastases, benign lesions, and normal vertebrae. ****P < 0.0001.

The HUs of benign lesions were higher than those of metastatic lesions and normal vertebrae; the HUs of metastatic lesions were higher than those of normal vertebrae; and the difference in HUs among the three groups was statistically significant (*P* < 0.0001). Metastatic lesions had larger volumes than benign lesions and normal vertebrae (*P* < 0.0001), but there was no statistically significant difference between the volumes of benign lesions and normal vertebrae (*P* = 0.1309) (Table 2, Figure 2).

**Table 2:** Number of metastatic bone lesions, benign bone lesions, and normal vertebrae as well as their SUVmax, HUs, and volume.

	**Metastatic lesions**	**Benign lesions**	**Normal vertebrae**
Number	252	140	199
SUVmax (mean ± SD)	23.85 ± 14.34	9.67 ± 7.47	6.19 ± 1.46
Median of SUVmax	19.86	8.21	6.03
Min. of SUVmax	4.74	3.49	2.4
Max. of SUVmax	75.33	66.27	11.62
Median HUs	244.1	393.5	159.19
Median volume (cm^3^)	8.37	2.61	3.76

SUVmax was measured in g/ml of body weight (BW). The results are presented as the mean ± SD. SUVmax, the maximum standardized uptake value; SD, the standard deviation; Minimum (Min.), the smallest value; Maximum (Max.), the highest possible value.

The volume unit is cm^3^. Bone density values were measured in Hounsfield units (HUs).

### 3.3. SUVmax discrimination for bone metastases

**Figure 3:**
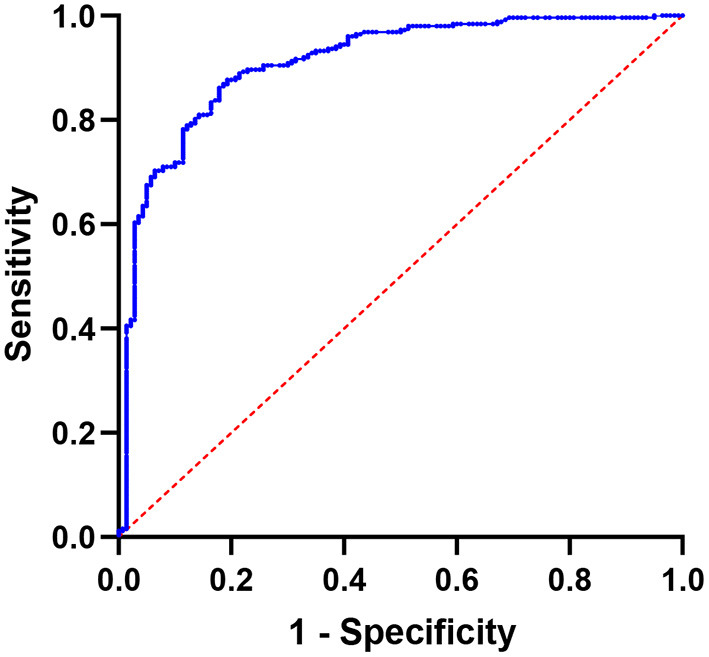
Using SUVmax, a receiver operating characteristic (ROC) curve was created to differentiate benign bone lesions from bone metastases; the area under the curve is 0.9097 (95% CI 0.8786–0.9407).

Figure 3 depicts the results of SUVmax ROC curve analyses. The SUVmax area under the curve value was 0.9097 (95% CI: 0.8786–0.9407, *P* < 0.0001). The optimal cutoff value for distinguishing hotspots of patients with bone metastases from those of patients with benign lesions in SPECT/CT was 11.10, with a sensitivity of 87.70% and a specificity of 80.71%.

### 3.4. SUVmax, HUs, and volume differences between metastatic lesions and four different CT features

Table 3 shows the number and percentage of total bone metastases for osteoblastic, osteolytic, mixed, and CT-negative metastatic lesions. The most common CT type of bone metastases in patients with lung adenocarcinoma was mixed lesions.

**Table 3:** The number, SUVmax values, HUs, and volume of bone metastatic lesions that were osteoblastic, osteolytic, mixed, or CT-negative.

	**Osteoblastic lesions**	**Osteolytic lesions**	**Mixed lesions**	**CT-negative lesions**
Number (%)	63 (25%)	53 (21.03%)	108 (42.86%)	28 (11.11%)
SUVmax	29.16 ± 16.63	15.79 ± 5.57	26.62 ± 14.97	16.51 ± 6.93
Median SUVmax	23.87	13.84	22.21	14.95
Min. SUVmax	7.23	4.74	7.18	8.18
Max. SUVmax	75.33	29.60	74.95	35.94
Median HUs	419.44	87.00	260.07	205.66
Median volume (cm^3^)	6.70	8.77	8.48	7.47

The number preceding % represents the percentage of all bone metastases. SUVmax was measured in g/ml of body weight (BW). The results are presented as the mean ± SD.

SUVmax, the maximum standardized uptake value; SD, the standard deviation; Minimum (Min.), the smallest value; Maximum (Max.), the highest possible value; Hus, Hounsfield units.

The volume unit is cm^3^.

**Figure 4:**
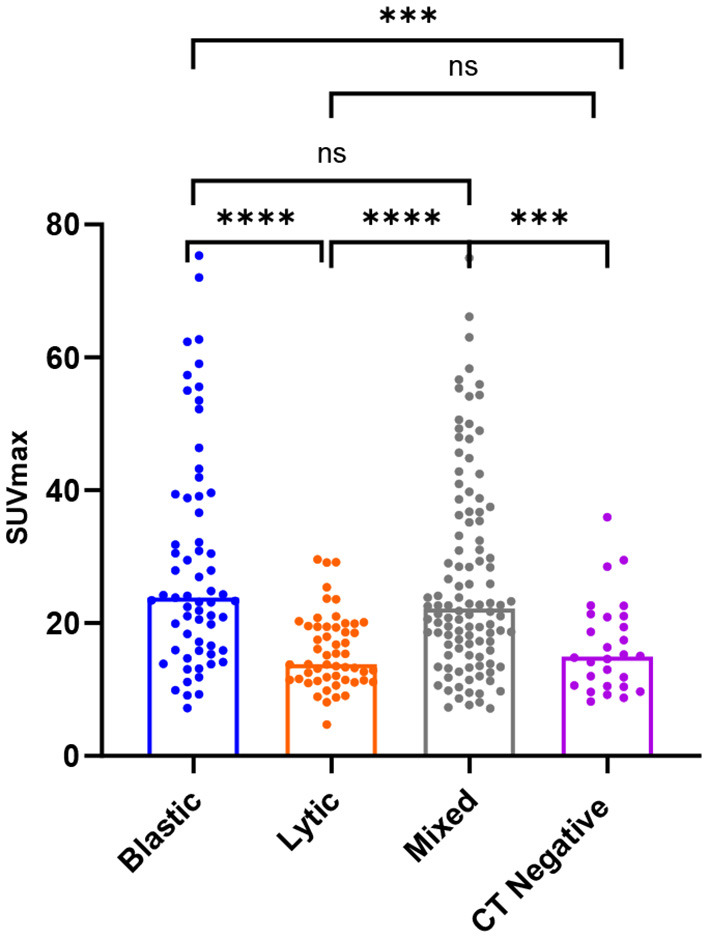
Box plots of all lesions in relation to the SUVmax (g/ml) for osteoblastic, osteolytic, mixed, and CT-negative metastatic lesions. ****P < 0.0001. ***P = 0.0003 and 0.002. *P* > 0.9999 is denoted by ns.

SUVmax was higher in osteoblastic (29.16 ± 16.63) and mixed (26.62 ± 14.97) metastatic lesions than in osteolytic (15.79 ± 5.57) lesions, and it was statistically significant (*P* < 0.0001). The SUVmax of osteoblastic and mixed lesions was significantly higher than that of CT-negative (16.51 ± 6.93) lesions (*P*-values = 0.0003 and 0.002, respectively). There was no statistically significant difference between the SUVmax of osteoblastic and mixed lesions (*P* > 0.9999) and between the SUVmax of osteolytic and CT-negative lesions (*P* > 0.9999; Table 3, Figure 4).

The average HUs of osteoblastic lesions was significantly greater than that of osteolytic, mixed, and CT-negative lesions (*P* < 0.0001). The average HUs of osteolytic lesions was significantly lower than that of mixed and CT-negative lesions (*P* < 0.0001). However, there was no statistically significant difference in HUs between mixed and CT-negative lesions (*P* > 0.9999). The volumes of the four different CT types of metastases did not differ statistically (*P* > 0.05).

### 3.5. SUVmax discrimination accuracy for CT-negative bone metastatic lesions

**Figure 5:**
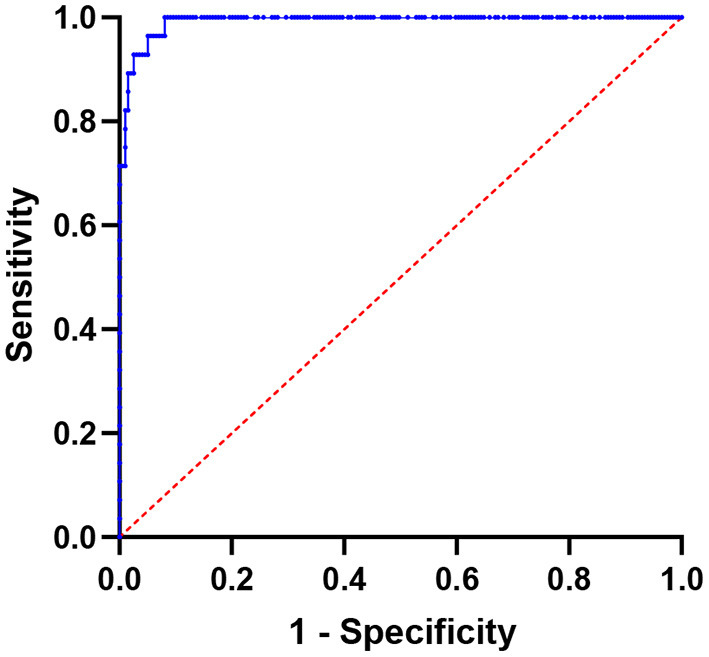
Using SUVmax, a receiver operating characteristic (ROC) curve was created to differentiate lesions between normal vertebrae and CT-negative bone metastases; the area under the curve is 0.9923 (95% CI 0.9839–1.000).

**Figure 6:**
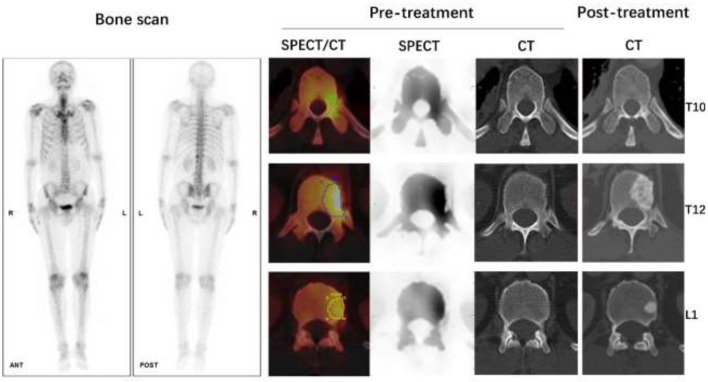
Male, 62 years old, CT guided biopsy of a tumor in the lower lobe of the right lung revealed a moderately differentiated adenocarcinoma, with an EGFR 19 mutation. Before treatment, a full-body bone scan revealed suspicious metastasis of the T10, T12, and L1 vertebrae. The SUVmax of lesions on T10, T12, and L1 vertebrae were 9.71, 14.80, and 10.52 g/ml, respectively, according to quantitative SPECT/CT. But the CT scan of these vertebrae on pre-treatment images showed no abnormal bone density changes. After 4 months of Almonertinib therapy, a post-treatment CT scan revealed increased bone mineral density in the high uptake area of SPECT images, confirmed bone metastasis of these vertebrae.

The area under the ROC for distinguishing between CT-negative metastatic lesions and normal vertebrae was 0.9923 (95% CI: 0.9839–1.000; *P*-value < 0.0001; *P*-value < 0.0001). The SUVmax at the cutoff value of 8.135, with a sensitivity of 100.00% and a specificity of 91.96%, can be used to differentiate CT-negative bone metastatic lesions (Figures 5, 6).

### 3.6. Linear correlation between SUVmax and HUs and between SUVmax and volume

**Figure 7:**
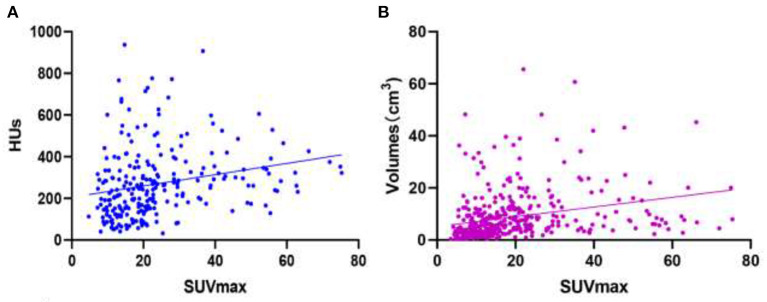
Images of linear correlation scatter plots. **(A)** Correlation between SUVmax and HUs from SPECT data for all bone metastatic lesions [*r* value = 0.2326 (95% CI 0.1123–0.3463), *P*-value = 0.0002]. **(B)** Correlation between SUVmax and volumes (cm^3^) from SPECT data for all bone lesions [*r* value = 0.2772 (95% CI 0.1832–0.3662), *P*-value < 0.0001].

SUVmax has a weak positive linear correlation with HUs for all bone metastatic lesions [*r* value = 0.2326 (95% CI: 0.1123–0.3463); *P*-value = 0.0002; Figure 7A]. SUVmax had a weak positive linear correlation with lesion volume for all bone lesions, including benign and metastatic lesions [*r* value = 0.2772 (95% CI 0.1832–0.3662); *P*-value < 0.0001; Figure 7B].

## 4. Discussion

Although body size, renal function, skeletal disease extent, and post injection acquisition time may affect SPECT/CT values, the skeletal quantification bone SPECT/CT has the potential to serve as a good biomarker of osteoblastic metabolism (14, 21). Previous studies (14, 16, 19) investigated the SUVs cutoff value to distinguish bone metastases from benign bone lesions. Yiqiu Zhang's and Flavian Tabotta's studies showed that SUVmax had greater accuracy than the average SUV (SUVave or SUVmean) in distinguishing bone metastasis from benign lesions (14, 19). Therefore, in the present study, we chose SUVmax as the only index to distinguish different lesions. To the best of our knowledge, few studies have analyzed SUVmax in bone lesions derived from patients with lung adenocarcinoma. Although the diagnoses of bone metastases in patients with lung adenocarcinoma mainly depend on the characteristics of CT images, the SUVmax of lesions obtained from quantitative SPECT/CT can be important when the CT findings of the bone lesions are atypical or there are high-uptake bone lesions without obvious CT abnormalities. We therefore aimed to explore the probable SUVmax cutoff value of bone metastases in patients with lung adenocarcinoma, especially the SUVmax cutoff value of CT-negative lesions, and analyze the SUVmax level of different CT characteristic bone metastases in patients with lung adenocarcinoma and factors that may affect SUVmax.

The SUVmax cutoff value for distinguishing bone metastases from benign bone lesions in this study was 11.10. This cutoff value was lower than that reported in previous studies, which included either all or some patients with prostate cancer (14, 16, 19). The reason for the lower SUVmax cutoff value in this study may be the lower SUVmax of bone metastases in patients with lung adenocarcinoma than in patients with prostate cancer. A study by Flavian Tabotta, which included 264 prostate cancer bone metastases (mean SUVmax 34.6 ± 24.6) and 24 spinal and pelvic osteoarthritic lesions (mean SUVmax 14.2 ± 3.8), showed an SUVmax cutoff of 19.5 g/ml for distinguishing bone metastases from osteoarthritic lesions (19). A study by Mohd Fazrin showed that the cutoff SUVmax value of ≥20 had a sensitivity of 73.8% and a specificity of 85.4% in differentiating bone metastases (mean SUVmax 36.64 ± 24.84) from degenerative joint disease (mean SUVmax 12.59 ± 9.01) in patients with prostate cancer. Bone metastases of prostate cancer trigger an important osteoblastic reaction and substantially accumulate ^99m^Tc-2,3-dicarboxy propane1,1-diphosphonate (^99m^Tc-DPD) or ^99m^Tc-MDP (19, 22), so the osteoblastic metastases have a higher SUVmax than that reported in our study that included patients with lung adenocarcinoma (mean SUVmax 23.85 ± 14.34). Even the osteoblastic metastases of lung adenocarcinoma in our study had a lower SUVmax (mean SUVmax 29.16 ± 16.63) than that of osteoblastic metastases in patients with prostate cancer in other studies. Moreover, this study also included 21.03% osteolytic (mean SUVmax 15.79 ± 5.57), 42.86% mixed (mean SUVmax 26.62 ± 14.97), and 11.11% CT-negative (mean SUVmax 16.51 ± 6.93) bone metastatic lesions, which had a lower SUVmax.

Another reason for the lower SUVmax cutoff value in this study may be the lower SUVmax of benign lesions (mean SUVmax 9.67 ± 7.47). The mean age (58.38 ± 9.92) of patients in this study was relatively lower than that in other studies on patients with prostate cancer (mean age 74 ± 10 years and 70.4 ± 7.4 years) (17, 19). During the aging process, changes occur in the extracellular matrix in the intervertebral disks, which result in narrowing of the joint space, nerve impingement, and instability of the joint. Consequent inflammation and remodeling of the bone tissue lead to calcification of the disc and formation of bony spurs or osteophytes (23). Therefore, degenerative lesions will be more obvious and may have higher SUVmax in older patients than in younger patients in the present study. Also, for all bone lesions, SUVmax had a weak linear correlation with volumes of the lesions in this study. This result was inconsistent with that of Fatin Halim's phantom research that showed at a sphere-to-background ratio of 1:4 with a high activity concentration, the SUVmax increased with an increase in sphere diameter (24). Therefore, with a relatively small volume of benign lesions in this study, SUVmax may be underestimated because of the partial volume effect.

In patients with lung adenocarcinoma, high-uptake lesions may be detected on SPECT, without obvious abnormal changes on CT. The diagnosis of such lesions is extraordinarily difficult. In the present study, we attempted to determine the likely SUVmax cutoff value of CT-negative metastatic lesions in patients with lung adenocarcinoma. A previous study on bone metastases from breast cancer revealed that the sensitivity of bone scans for detecting different CT types of bone metastasis was 100% (21/21) for mixed lesions, 94% (15/16) for osteoblastic lesions, 90% (28/31) for osteolytic lesions, and 70% (14/20) for CT-negative lesions (25). SPECT/CT may improve the diagnostic sensitivity of CT-negative lesions. We chose normal vertebrae as the comparative sample of normal bone because normal vertebrae have relatively stable uptake, as shown in previous studies. A study by Mohd Farina (16) showed that the mean SUVmax of 234 normal vertebrae was 7.08 ± 1.97 in patients with prostate cancer. In another study, the mean SUVmax of 120 vertebrae in the no-treatment breast cancer group was 5.37 ± 2.81 (26). The mean SUVmax of 199 normal vertebrae was 6.19 ± 1.46 in our group of patients with lung adenocarcinoma. Moreover, in the present study, most of the CT-negative bone metastatic lesions were located in vertebrae (78.6%, 22/28). The mean SUVmax of CT-negative bone metastatic lesions was 16.51 ± 6.93, when using a cutoff value of 8.135, CT-negative bone metastatic lesions may be discriminated with a sensitivity of 100.00% and a specificity of 91.96%. With quantitative SPECT/CT, when focal high-uptake lesions with an SUVmax of >8.135 are detected, bone metastasis should be highly suspected and further examination with MRI or PET/CT is recommended.

Despite having a lower SUVmax than studies involving patients with prostate cancer, the SUVmax of bone metastases in this study (SUVmax 23.85 ± 14.34, 4.74–75.33) was nearly the same as that in a study by Zhang et al. (14), which included 30 patients with lung cancer and 21 patients with other cancers. In their study, the SUVmax of metastases was 24.77 ± 16.32 (3.90–92.61). When referring to the uptake of ^99m^Tc-MDP in different kinds of CT features, Guray Gurkan's study, which included different cancer patients and used BS, showed that the mean ROImax (maximum lesion to normal bone count ratio on BS) of osteoblastic bone lesions (6.42 ± 4.22) and mixed metastases (6.32 ± 4.03) was higher than that of osteolytic lesions (5.33 ± 3.60), but there was no significant difference in the mean ROImax in osteolytic, osteoblastic, and mixed lesions (*P* > 0.05) (27). However, in this study using quantitative SPECT/CT, the SUVmax of osteoblastic (29.16 ± 16.63) and mixed (26.62 ± 14.97) lesions was significantly higher than that of osteolytic (15.79 ± 5.57) and CT-negative (16.51 ± 6.93) lesions (*P* < 0.05). A previous study revealed that osteoblastic lesions had significantly higher HUs than osteolytic and mixed lesions (*P* < 0.01) (27). In our study, we discovered that SUVmax had a weak positive linear correlation with HUs for all bone metastatic lesions. As a result, for osteoblastic and mixed lesions that had higher HUs, the SUVmax of osteoblastic and mixed metastatic lesions was significantly higher than that of osteolytic and CT-negative lesions with lower HUs.

The present study had some limitations. It was conducted retrospectively, and the results may have been influenced by the criteria used to patient selection, for example, some of the patients in this study received the SPECT/CT acquisition delayed by some uncontrollable reasons. The acquisition time may influence the uptake of the normal vertebrae and bone metastatic lesions (21). We will set fixed acquisition time and explore the time influence for the SUVmax in bone metastatic lesions in our future prospective research. Furthermore, majority of the patients in this study lacked histological confirmation of bone metastases; however, all lesions were followed up for more than 0.5 year, and the initial imaging results were confirmed by re-examination of whole-body bone scans and SPECT/CT, CT, MR, or PET/CT scans. Cases that lacked follow-up data were excluded from the study. Some of the patients (47, 40.9%) in the present study had more than six metastatic lesions. There is a possibility that the diagnostic value of SUVmax might be more valuable for patients with less bone metastasis. However, this was a preliminary exploratory study of SUVmax uptake in bone metastases in patients with lung adenocarcinoma. The SUVmax changes of bone metastatic lesions after treatment may have more guidance value for clinical practice of lung adenocarcinoma patients; this is what we will study in the future. Because of the relatively small number of patients in this study, we did not perform a sub analysis on gender-related cutoff values for distinguishing CT-negative bone metastases from normal vertebrae. We will explore the influence of gender with more cases in a future study.

## 5. Conclusion

SUVmax of quantitative SPECT/CT is a useful index for distinguishing benign bone lesions from bone metastases in patients with lung adenocarcinoma, particularly in the diagnosis of CT-negative bone metastases. However, other factors, such as HUs and volume, which may affect the SUVmax, should still be considered.

## Data availability statement

The raw data supporting the conclusions of this article will be made available by the authors, without undue reservation.

## Ethics statement

Written informed consent was obtained from the individual(s) for the publication of any potentially identifiable images or data included in this article.

## Author contributions

LL, XW, and RZ contributed to the conception and design of the study. LL, RF, YZ, KY, and JG organized the database. ML and JG carried out data statistics and analysis. LL wrote the manuscript. RZ, JG, XW, and ML revised the manuscript. All authors have read and approved the final manuscript.
