# Supplementary material for: Skeletal standardized uptake values obtained using quantitative SPECT/CT for the detection of bone metastases in patients with lung adenocarcinoma
Source: Front Med (Lausanne). 2023 Feb 2;10:1119214. doi: 10.3389/fmed.2023.1119214 (PMC9931902; doi:10.3389/fmed.2023.1119214)
Supplement: Supplementary file 1 [file Table_1.DOCX]

**Supplementary Table: The SUVmax and HU of normal vertebrae in males and females**

|  | **Normal vertebrae of males** | **Normal vertebrae of females** | **P value** |
| --- | --- | --- | --- |
| **Number** | 83 | 116 |  |
| **SUVmax（Mean ± SD)** | 6.13 ± 1.26 | 6.24 ± 1.58 |  |
| **Median of SUVmax** | 6.03 | 6.08 | P = 0.728 |
| **Min. of SUVmax** | 3.45 | 2.40 |  |
| **Max. of SUVmax** | 9.49 | 11.62 |  |
| **Mean ± SD, Median HU** | 174.38 ± 66.89, 157.46 | 166.57 ± 76.98, 161.00 | P = 0.455 |
